# Supplementary material for: SARS-CoV-2 Surveillance in Belgian Wastewaters
Source: Viruses. 2022 Sep 2;14(9):1950. doi: 10.3390/v14091950 (PMC9506219; doi:10.3390/v14091950)
Supplement: Supplementary file 1 [file viruses-14-01950-s001.zip › viruses-1830482-supplementary.pdf]

viruses

Article – Supplementary Materials

SARS-CoV-2 Surveillance in Belgian Wastewaters

Supplementary Materials

Table S1. Covered population, province, region and laboratory corresponding to the wastewater treatment plants included in the national surveillance. The identification numbers (id.) are localized on the Belgian map in Figure S1.

Id.	Treatment Plant	Population	Provinces	Region	Laboratory
1	Aalst	102,800	Oost-Vlaanderen	Flanders	Sciensano
2	Aartselaar	68,031	Antwerpen	Flanders	UAntwerpen
3	Amay	47,038	Liège	Wallonia	E-BIOM
4	Antwerpen-Noord	76,949	Antwerpen	Flanders	UAntwerpen
5	Antwerpen-Zuid	215,704	Antwerpen	Flanders	UAntwerpen
6	Arlon	19,440	Luxembourg	Wallonia	E-BIOM
7	Basse Wavre (Dyle)	78,290	Brabant Wallon	Wallonia	E-BIOM
8	Beersel	78,540	Vlaams-Brabant	Flanders	Sciensano
9	Boom [1]	37,846	Antwerpen	Flanders	UAntwerpen
10	Brugge	200,401	West-Vlaanderen	Flanders	Sciensano
11	Bruxelles-Nord/Brussel-Noord	1,045,863	Brussels	Brussels	Sciensano
12	Bruxelles-Sud/Brussel-Zuid	311,866	Brussels	Brussels	Sciensano
13	Dendermonde	87,633	Oost-Vlaanderen	Flanders	UAntwerpen
14	Destelbergen	63,771	Oost-Vlaanderen	Flanders	Sciensano
15	Deurne	213,048	Antwerpen	Flanders	UAntwerpen
16	Froyennes	34,710	Hainaut	Wallonia	E-BIOM
17	Genk	73,364	Limburg	Flanders	UAntwerpen
18	Gent	247,550	Oost-Vlaanderen	Flanders	Sciensano
19	Grimbergen	120,627	Vlaams-Brabant	Flanders	Sciensano
20	Harelbeke	125,230	West-Vlaanderen	Flanders	Sciensano
21	Hasselt	81,988	Limburg	Flanders	UAntwerpen
22	Houthalen-Centrum	22,357	Limburg	Flanders	UAntwerpen
23	Leuven	137,365	Vlaams-Brabant	Flanders	Sciensano
24	Liedekerke	112,933	Vlaams-Brabant	Flanders	Sciensano
25	Liège (Grosses Battes) [2]	27,934	Liège	Wallonia	E-BIOM
26	Liège Oupeye	253,780	Liège	Wallonia	E-BIOM
27	Liège Sclessin	142,239	Liège	Wallonia	E-BIOM
28	Marche-en-Famenne	8,633	Luxembourg	Wallonia	E-BIOM
29	Marchienne-au-Pont	52,017	Hainaut	Wallonia	E-BIOM
30	Mechelen-Noord	116,363	Antwerpen	Flanders	UAntwerpen
31	Menen	70,041	West-Vlaanderen	Flanders	Sciensano
32	Montignies-sur-Sambre	123,576	Hainaut	Wallonia	E-BIOM
33	Mornimont	34,140	Namur	Wallonia	E-BIOM
34	Mouscron versant Espierres	21,179	Hainaut	Wallonia	E-BIOM
35	Namur-Brumagne	82,175	Namur	Wallonia	E-BIOM
36	Oostende	153,401	West-Vlaanderen	Flanders	Sciensano

37	Roeselare	82,137	West-Vlaanderen	Flanders	Sciensano
38	Sint-Niklaas	55,038	Oost-Vlaanderen	Flanders	UAntwerpen
39	Soumagne [3]	7,955	Liège	Wallonia	E-BIOM
40	Tessenderlo	55,546	Limburg	Flanders	UAntwerpen
41	Turnhout	44,524	Antwerpen	Flanders	UAntwerpen
42	Vallee du Hain (l'Orchis)	57,050	Brabant Wallon	Wallonia	E-BIOM
43	Wasmuel	157,342	Hainaut	Wallonia	E-BIOM
44	Wegnez [2]	78,244	Liège	Wallonia	E-BIOM

[1] The sampling in Boom was interrupted and replaced by Tessenderlo on 01/01/2021. [2] The sampling of Wegnez and Liège (Grosses Battes) was interrupted in July 2021 as major flooding events damaged the treatment plants. [3] The sampling of Soumagne started on 24/08/2021.

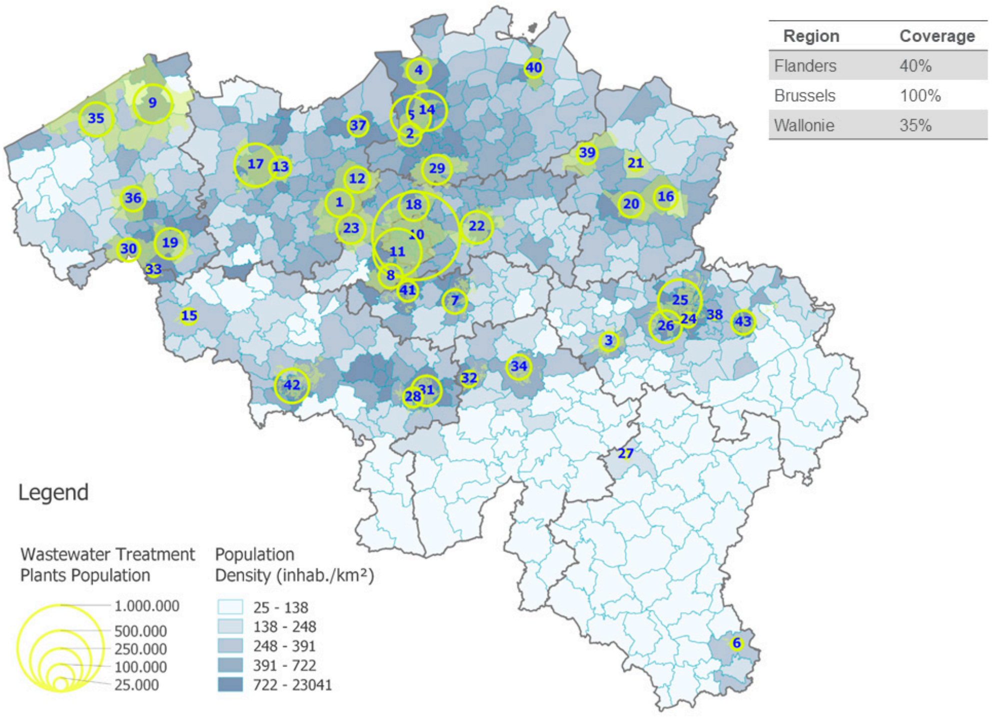

Figure S1. The localization of the treatment plants included in the wastewater surveillance and their identification number as described in Table S1. The population located in the areas covered by the wastewater treatment plants are highlighted in yellow and the population density for each municipality is indicated with the blue scale.

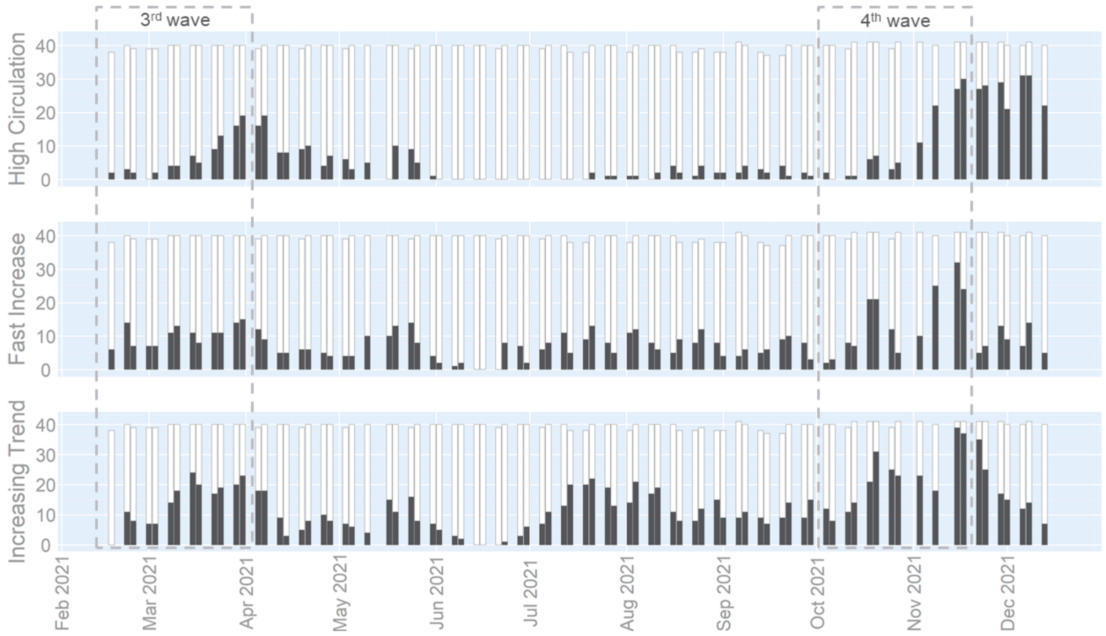

Figure S2. Evolution of the number of areas for which the wastewater indicators are positives. The indicators are computed on the viral load per capita (SARS-CoV-2 RNA copies/day/100 k inhab.) with the 3rd wave as reference period (15/02/2021–01/04/2021): High Circulation, Fast Increase, and Increasing Trend. The number of areas for which each indicator is positive or not is indicated by black and white bars, respectively. The total number may be lower than 42 when technical issues prevented samples to be taken for some of the wastewater treatment plants.

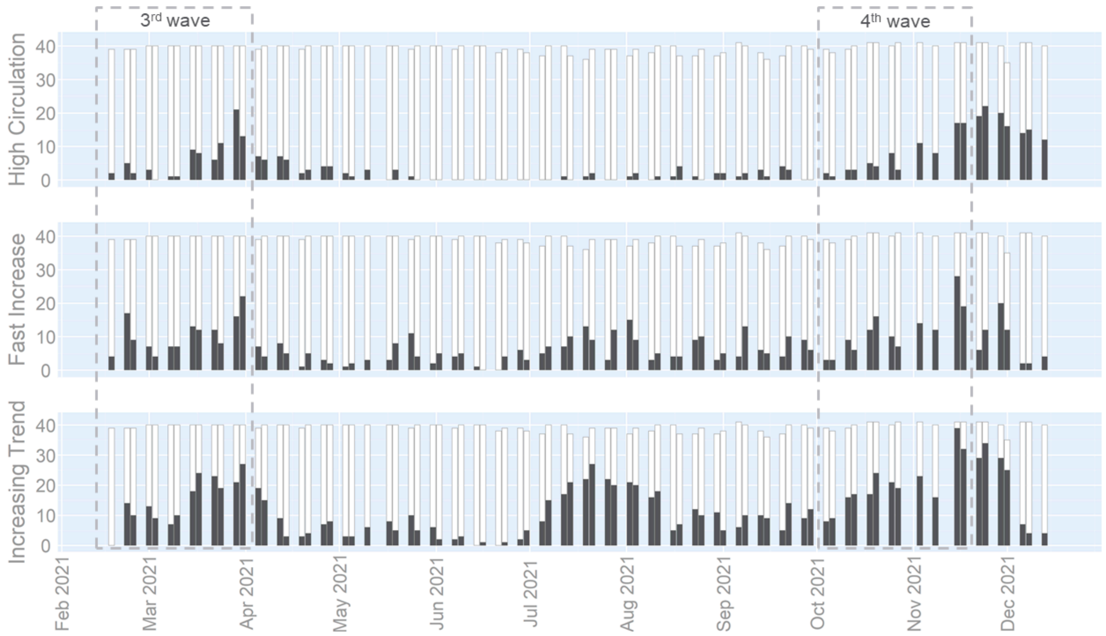

Figure S3. Evolution of the number of areas for which the wastewater indicators are positives. The indicators are computed on the viral to faecal ratio (SARS-CoV-2 RNA gene copies/PMMoV RNA gene copies) with the 3rd wave as reference period (15/02/2021–01/04/2021): High Circulation, Fast Increase, and Increasing Trend. The number of areas for which each indicator is positive or not is indicated by black and white bars, respectively. The total number may be lower than 42 when technical issues prevented samples to be taken for some of the wastewater treatment plants.

Table S2. Spearman correlation coefficients computed for the three combinations of viral concentration of targeted SARS-CoV-2 gene fragments (N1 vs. N2, N1 vs. E, N2 vs. E) grouped by month between February 2021 and December 2021. The number (N) of viral concentration measurements

included in the analysis is shown. Data of the 42 treatment plants were used and were, thus, not aggregated at a national level. All correlations were significant ($p < 0.0001$).

**Month**	**N**	**N1 vs. N2**	**N1 vs. E**	**N2 vs. E**
March 2021	400	0.93	0.86	0.85
April 2021	318	0.95	0.80	0.89
May 2021	319	0.93	0.87	0.87
June 2021	360	0.92	0.88	0.83
July 2021	315	0.96	0.87	0.90
August 2021	351	0.96	0.91	0.91
September 2021	339	0.98	0.95	0.95
October 2021	323	0.98	0.94	0.95
November 2021	286	0.97	0.89	0.92
December 2021	162	0.98	0.89	0.88
